# Exploring the Potential of Venom from *Nasonia vitripennis* as Therapeutic Agent with High-Throughput Screening Tools

**DOI:** 10.3390/toxins7062051

**Published:** 2015-06-03

**Authors:** Ellen L. Danneels, Ellen M. Formesyn, Dirk C. de Graaf

**Affiliations:** Laboratory of Molecular Entomology and Bee Pathology, Ghent University, S2 Krijgslaan 281, B-9000 Ghent, Belgium; E-Mails: ellen.formesyn@gmail.com (E.M.F.); dirk.degraaf@ugent.be (D.C.G.)

**Keywords:** *Nasonia*, venom, therapeutic, reporter array, PCR array, NF-κB

## Abstract

The venom from the ectoparasitoid wasp *Nasonia vitripennis* (Hymenoptera: Pteromalidae) contains at least 80 different proteins and possibly even more peptides or other small chemical compounds, demonstrating its appealing therapeutic application. To better understand the dynamics of the venom in mammalian cells, two high-throughput screening tools were performed. The venom induced pathways related to an early stress response and activated reporters that suggest the involvement of steroids. Whether these steroids reside from the venom itself or show an induced release/production caused by the venom, still remains unsolved. The proinflammatory cytokine IL-1β was found to be down-regulated after venom and LPS co-treatment, confirming the anti-inflammatory action of *N. vitripennis* venom. When analyzing the expression levels of the NF-κB target genes, potentially not only the canonical but also the alternative NF-κB pathway can be affected, possibly explaining some counterintuitive results. It is proposed that next to an NF-κB binding site, the promoter of the genes tested by the PCR array may also contain binding sites for other transcription factors, resulting in a complex puzzle to connect the induced target gene with its respective transcription factor. Interestingly, *Nasonia* venom altered the expression of some drug targets, presenting the venom with an exciting therapeutical potential.

## 1. Introduction

Hymenopteran parasitoids develop at the expense of other arthropods, ultimately killing their host. The ectoparasitoid wasp *Nasonia vitripennis* (Hymenoptera: Pteromalidae) preferably parasitizes pupae from flesh flies (Sarcophagidae) and blow flies (Calliphoridae). After locating a suitable host, the female wasp injects venom inside the fly pupa and lays her eggs in the space between the pupa and the puparium. The injection of this complex mixture of venom compounds prepares the host to present the best possible environment for the wasp offspring to survive. Host physiology is altered, in which the host development is arrested, its immune system is suppressed, and host metabolism is modified so that it is synchronized with the development of parasitoid larvae.

The venom from *N. vitripennis* is known to contain (at least) 80 different proteins [1,2], and possibly even peptides and other bio-molecules. Over the past century, natural products (NPs) have been the source of inspiration for the majority of FDA approved drugs. This is highlighted by the fact that nearly 50% of all drugs in clinical use are of natural product origin [3]. These interesting chemicals are derived from the phenomenon of biodiversity in which the interactions among organisms and their environment formulate the diverse complex chemical entities within the organisms that enhance their survival and competitiveness [4]. The therapeutic areas of infectious diseases and oncology have benefited from the complex molecular scaffolds found in NPs of which the chemical diversity is unmatched by synthetic molecules. Animal venoms are a rich source of NPs that have evolved high affinity and selectivity for a diverse range of biological targets, especially membrane proteins such as ion channels, receptors, and transporters. Therefore, venomics has emerged as an important addition to modern drug discovery efforts [5]. Snake venom is a treasure house of toxins that contributes significantly to the treatment of many medical conditions and presents a great potential as an anti-tumor agent [6]. The venom and its constituents from honey bees have many therapeutic applications ranging from anti-arthritis and pain-releasing to anti-cancer effects [7]. Venoms from parasitoid wasps contain a staggering amount of toxins, and because they can manipulate cell physiology in diverse ways [8,9,10], their therapeutic potential is interesting to investigate.

Although the natural hosts of *N. vitripennis* wasps are insect pupae, one might wonder how the venom-induced physiological alterations would translate to a mammalian system. The concept “bugs as drugs” emphasizes the interest in mining insects for medicinal drugs [11]. With the intent to explore a possible medicinal future, we performed a wide screening of the effects of this *Nasonia* venom on mammalian cellular signaling with high-throughput arrays that are designed for use with mammalian cells. However, to further investigate the specific interaction mechanism, *Drosophila melanogaster* could be used as a model system, which is less of a leap from the *Nasonia-*host insect system.

Cell-based assays provide a high-performance tool due to their exceptional sensitivity, reproducibility, specificity, and signal-to-noise ratio, for assessing the functions of natural products under physiological cellular conditions. By screening multiple pathway activities simultaneously, relevant pathways for further analysis can quickly be identified. Therefore, possible regulation by the complete venom mixture of 45 reporters that represent transcription factors (TFs) that play a central role in regulating gene expression, orchestrating a host of cellular processes, and are associated with many human diseases were investigated using a reporter array (Cignal™ 45-Pathway Reporter Array, SABiosciences, Frederick, MD, USA). Of the 45 pathways, four main research areas were targeted, including cancer, immunity, development, and toxicology. By reverse transfecting human embryonic kidney (HEK293T) cells into multi-pathway reporter arrays, the activity of 45 pathways was screened upon *N. vitripennis* venom treatment. Transcriptional activity is monitored by the dual luciferase technology that allows for quantification of the degree of activation of each particular signaling pathway in a 96 well format By analyzing the effects of the venom on multiple cellular signaling pathways, new directions for further investigations with possible biomedical application could be appointed.

Recently, *N. vitripennis* venom was shown to exert a suppressive action on the nuclear factor kappa-light-chain-enhancer of activated B cells (NF-κB) pathway in murine macrophages [8]. This important TF regulates a large number of target genes involved in multiple cellular processes including inflammation, immunity, and stress responses [12]. Dysregulation of this signal transduction pathway has been associated with inflammatory or autoimmune diseases [13] and cancer [14]. Previous investigations showed that lipopolysaccharide (LPS)-induced NF-κB activation in Raw264.7 macrophages resulted in an inhibition of the inflammatory response when the cells were incubated with *N. vitripennis* venom. By further investigating the interruption of this crucial immune pathway by the venom, it appeared that next to the suppression of the NF-κB cascade also the mitogen-activated protein kinase (MAPK) and glucocorticoid receptor (GR) signaling pathways were affected. Therefore, in order to fully explore venom activities on intracellular signaling after an immune activation, TNFα-induced HEK293T cells were incubated with *N. vitripennis* venom and also analyzed with the reporter array.

In 1999, Pahl listed over 150 target genes known to be expressed by the active NF-κB transcription factor [15]. To date, this list has been extended by more than 250 extra investigated NF-κB target genes and even more than 300 genes are predicted by computer-based methods to have composite NF-κB regulatory sites [16]. The majority of proteins encoded by NF-κB target genes participate in the host inflammatory and immune responses, which include cytokines and chemokines, as well as receptors required for immune recognition, proteins involved in antigen presentation, acute phase proteins, and cell adhesion molecules. Many of them are induced by exposure to a wide variety of bacteria, as well as hosts of viruses and their respective products. NF-κB, however, is involved in the control of the transcription of many genes whose functions extend beyond the immune response, but are involved in more general stress responses [17]. Various physiological stress conditions such as liver regeneration and hemorrhagic shock can activate NF-κB [18,19]. Also physical stress, in the form of irradiation as well as oxidative stress to cells, induces NF-κB, that in turn activates a large variety of stress response genes [20]. In fact, NF-κB relays the information of an imminent stress and at the same time enacts a response by promoting the transcription of genes whose products alleviate the stress condition. The human body is also exposed to environmental hazards and therapeutic drugs, activating NF-κB that in turn activates its target genes including many cell surface receptors [21]. Several stimuli, among them the cytokine TNFα, can lead to NF-κB activation that exerts anti-apoptotic activities. On the other hand, there is ample evidence for apoptosis-promoting functions of NF-κB as well [22,23]. The nature of the apoptotic stimulus determines the pro- or anti-apoptotic function of NF-κB. Activation of NF-κB can lead to the transcriptional induction of various TF genes, even members of their own Rel/NF-κB/IκB family.

The enormous amount of NF-κB target genes can be categorized in the different groups mentioned above, which has been done for the 84 tested genes in the NF-κB Signaling Targets PCR Array. To gain broader understanding of the effects of *N. vitripennis* venom on the immune response, the expression of these key genes responsive to NF-κB signal transduction were analyzed after incubation of Raw264.7 macrophages with this venom mixture. Additionally, alterations of specific NF-κB signaling target genes with a role in inflammatory diseases and oncology could hint at potential therapeutic lead compounds present in the venom.

## 2. Results and Discussion

### 2.1. Effect of Venom on Mammalian Intracellular Signaling

To investigate the effects of *N. vitripennis* venom on mammalian intracellular signaling, we screened a wide range of signaling pathways for their regulation after 8 h incubation with venom. We utilized a commercially available array (Cignal™ 45-Pathway Reporter Array, SABiosciences, Frederick, MD, USA) on HEK293T cells. These cells are easily and efficiently transfected with PEI transfection reagent. By comparison of two reporter constructs, the pathway-focused TF-responsive Firefly luciferase reporter and the constitutively expressing Renilla luciferase construct, activated pathways are identified. This dual luciferase technology allows for quantification of the degree of activation of each particular signaling pathway in a 96 well format. Since the venom from *N. vitripennis* was found to suppress the NF-κB pathway in fibrosarcoma cells when induced with tumor necrosis factor alpha (TNFα) [8], we also used TNFα-induced HEK293T cells in this reporter array to investigate the effects of the venom on intracellular signaling when the immune system in these cells is activated. All conditions were performed in four replicates and internal positive and negative controls on all plates were fulfilled.

The significant values with a fold change higher than 2-fold are summarized in Table 1 for the following three comparisons: venom-treated *versus* untreated cells, TNFα-treated *versus* untreated cells, and venom and TNFα co-treated *versus* TNFα-treated cells. The incubation of cells with *N. vitripennis* venom caused an increased activation of four reporters compared to control cells: the AARE, LXR, MEF2 and RXR reporters. The reporter that showed the highest up-regulation was the amino acid deprivation (AARE) reporter which is known to be an early response upon stress [24]. Malnutrition, various pathological situations, and xenobiotic toxins are able to alter amino acid availability which can result in a deficit of certain amino acids and increased uncharged transfer RNAs (tRNAs). Following amino acid deprivation, the general control non-derepressible-2 (GCN2) kinase is activated upon detection of these accumulated free tRNAs and phosphorylates the translation initiation factor (eukaryotic initiation factor 2a), thereby attenuating protein synthesis. Since the endoplasmic reticulum (ER) regulates the production and oxidative folding of proteins, this response may prevent further accumulation of misfolded proteins and alterations in redox state [25]. Furthermore, phosphorylated eIF2a enhances the translation of several mRNAs, including ATF4 which is rapidly induced under cell-stress conditions, such as glutathione depletion and oxidative stress. ATF4 is an important regulator of several ER stress target genes, amino acid transporters and antioxidants thereby preventing further accumulation of reactive oxygen species (ROS) [26]. Interestingly, free amino acids (FAA) were found to be up-regulated in the natural host after parasitization by *N. vitripennis* [27]. They suggested that the larval parasitoids use these FAA as a source of direct nutrition. Whether both processes concerning the elevation of amino acids in mammalian cells and the insect hosts can be linked together still needs to be elucidated.

**Table 1:** Effect of *N. vitripennis* venom in HEK293T cells, either induced with TNFα or not, on the transcriptional activity of reporters of 45 different pathways. Fold regulation (FR) of all tested reporters are presented for 3 different comparisons. When *p* > 0.05, insignificant values are between brackets; when |FR| > 2, values are in bold.

Pathway reporters	FR TNFα-treated *versus* untreated	FR Venom-treated *versus* untreated	FR TNFα- and venom-treated *versus* TNFα-treated
AARE reporter	1.601	**4.081**	**4.555**
AR reporter	**−2.613**	(1.291)	**7.814**
C/EBP reporter	(1.274)	1.925	**2.507**
CRE reporter	1.779	(1.457)	**2.020**
E2F reporter	1.001	(1.012)	**3.560**
p53 reporter	(**2.569**)	(**3.867**)	**4.177**
EGR1 reporter	(−0.911)	(1.548)	**2.228**
HSR reporter	(−0.901)	(1.311)	**3.999**
GLI reporter	**−3.073**	(−0.240)	**7.556**
IRF1 reporter	−1.188	(1.287)	**2.568**
LXR reporter	(−0.342)	**2.201**	1.622
MEF2 reporter	−1.192	**2.023**	**2.849**
NF-κB reporter	**100.640**	(−1.272)	(1.145)
Oct4 reporter	**−2.336**	(−1.436)	**3.007**
PR reporter	**10.676**	(−0.126)	(1.718)
RARE reporter	(−0.702)	(−0.048)	**2.3887**
RXR reporter	(−0.370)	**2.539**	(1.379)

Two other reporters up-regulated by the venom are the LXR and the RXR reporter, that measure the transcriptional activity of the Liver X receptor and the Retinoid X receptor, respectively. The natural ligands of LXRs are oxygenated forms of cholesterol, while RXRs are activated upon binding with vitamin A, derivatives, and rexinoids. The regulation of both receptors through binding with small chemical compounds that can pass biological membranes, could imply that compounds other than proteins are present in the venom, which can migrate through membranes and bind directly to TFs. Today, nothing is known about such compounds in the venom of *N. vitripennis*, presenting us with an unexplored and unexploited source of potentially useful compounds for medicine to further investigate.

When HEK293T cells were induced for 8 h with TNFα, the activation of two reporters was significantly up-regulated, while three were down-regulated compared to control cells. The NF-κB reporter was no less than 100 times up-regulated, showing the strong activation of this immune cascade. When cells were co-incubated with TNFα together with venom from *N. vitripennis*, no significant alteration in transcriptional activity could be observed compared to cells simply induced with TNFα. In L929sA cells however, a significant inhibition of the NF-κB activation was noted by the venom [8]. This can possibly be explained by the use of different cell lines, HEK293T cells instead of L929sA cells, or other induction times, 8 h instead of 6 h.

When cells were incubated with TNFα together with venom, no less than 13 of the 45 tested pathways showed significant differential transcriptional activity, compared to cells that were only induced with TNFα. Interestingly, the AR and the GLI reporter, that show down-regulated transcriptional activity after TNFα treatment, have the highest up-regulated activity (more than seven times higher) when co-incubated with *N. vitripennis* venom. The androgen receptor (AR) is a nuclear receptor that is activated by binding androgenic hormones, testosterone, or dihydrotestosterone, again hinting at possible presence of small chemical compounds in the venom. The GLI reporter is designed to measure hedgehog signaling activity. Interestingly, the sonic hedgehog signaling pathway plays an important role in the development of cancer, specifically brain and skin cancer [28]. Analysis of the reporter array also revealed that several reporters, like the p53 and HSR reporter involved in stress responses were affected by the venom when co-incubated with TNFα for 8 h. In addition, microarray on parasitized insect hosts also revealed that transcripts involved in stress, cell death, detoxification, and the MAPK/JNK pathways were affected by the venom of *N. vitripennis* [29].

Other interesting reporters involved in inflammation and the immune response showed significant alterations in transcriptional activity after co-treatment of HEK293T cells with TNFα and *N. vitripennis* venom. The Interferon Regulatory Factor 1 (IRF1) reporter for instance, is a member of the interferon regulatory TF family and serves as a transcriptional activator of interferon alpha, beta, and gamma. IRF1 is known to regulate host defense against pathogens, tumor prevention, and development of the immune system. Accordingly, in the natural host, the venom is known to allow or even stimulate certain antimicrobial defenses of the host next to the expected interference of the venom with host melanization and coagulation responses [30].

The reporter arrays revealed that the venom from *N. vitripennis* has a wide-spread impact on multiple mammalian signaling cascades. However, it would be interesting to investigate what the effect would be on transcriptional activity when separate venom compounds would be used to induce the cells. Therefore, not only proteins or peptides, but also small chemical compounds like amines or steroids that are possibly present in the venom from *N. vitripennis*, should be isolated from the complete venom mixture and further investigated for their effects on the cellular signaling cascades that were found to be targeted by the complete venom.

### 2.2. Effect of Venom on NF-κB Signaling Targets

Since previous results on L929sA cells showed clear anti-inflammatory activity of the venom [8], we decided to dig deeper in the NF-κB regulating effects of the *N. vitripennis* venom. Therefore, a PCR array experiment was set up to evaluate the expression of NF-κB target genes under different conditions. We decided to use Raw264.7 macrophage cells that show activation of the NF-κB signaling cascade when stimulated with LPS. We treated these cells with *N. vitripennis* venom, together with or without LPS induction. Significant relative expression of 84 genes was evaluated by RT-qPCR as presented in Table S1. The genes that show a significant differential expression of more than 2-fold higher or lower in three different comparisons are summarized in Table 2 in which the NF-κB target genes are classified according to their properties and/or functions.

**Table 2:** Effect of *N. vitripennis* venom in Raw264.7 cells, either induced with lipopolysaccharide (LPS) or not, on NF-κB signaling targets. Fold regulation of all tested NF-κB signaling target genes are presented for 3 different comparisons. When *p* > 0.05, insignificant values are between brackets; when |FR| > 2, values are in bold. (Abb = abbreviation; FR = fold regulation).

NF-κB signaling target genes	Abb		FR LPS-treated *versus* untreated		FR venom-treated *versus* untreated		FR LPS- and venom-treated *versus* LPS-treated	
*Cytokines/chemokines and their modulators*								
Chemokine (C–C motif) ligand 22	Ccl22		**917.635**		(1.032)		(−1.532)	
Chemokine (C–C motif) ligand 5	Ccl5		**1254.881**		(2.107)		(1.750)	
Chemokine (C–C motif) receptor 5	Ccr5		1.625		(1.875)		(2.346)	
Chemokine (C–X–C motif) ligand 10	Cxcl10		**483.835**		(2.254)		(−1.025)	
Chemokine (C–X–C motif) ligand 3	Cxcl3		**111.806**		(3.484)		(34.595)	
Interleukin 15	Il15		(3.325)		(2.527)		**13.408**	
Interleukin 1α	Il1a		**1273.082**		(2.414)		(−1.242)	
Interleukin 1β	Il1b		**15,647.327**		**5.885**		**−4.392**	
Interleukin 1 receptor antagonist	Il1rn		**26.052**		(3.074)		(−1.145)	
Interleukin 6	Il6		**525.452**		(2.419)		−1.472	
Lymphotoxin A	Lta		**25.056**		(3.037)		(1.094)	
Tumor necrosis factor	Tnf		**36.399**		(−1.244)		−1.193	
*Immunoreceptors*								
CD40 antigen	Cd40		**68.505**		**9.933**		(4.098)	
CD80 antigen	Cd80		**3.113**		(2.621)		(2.723)	
CD83 antigen	Cd83		(3.545)		(2.949)		**29.395**	
Tumor necrosis factor receptor superfamily, member 1b	Tnfrsf1b		**22.445**		(1.469)		(1.708)	
*Proteins involved in antigen presentation*								
Complement component 3	C3		**3.651**		(2.035)		(1.175)	
Complement factor B	Cfb		**14.677**		−(1.007)		**−4.199**	
*Cell adhesion molecules*								
Intercellular adhesion molecule 1	Icam1		(1.405)		(1.212)		**17.631**	
Vascular cell adhesion molecule 1	Vcam1		(−1.148)		(1.387)		**3.481**	
*Acute phase proteins*								
Coagulation factor III	F3		**41.407**		(4.466)		(−1.979)	
*Stress response genes*								
NAD(P)H dehydrogenase, quinone 1	Nqo1		(−1.247)		**24.637**		(3.014)	
Prostaglandin-endoperoxide synthase 2	Ptgs2		**698.790**		(1.715)		(−1.605)	
Superoxide dismutase 2, mitochondrial	Sod2		**4.919**		−1.052		(1.301)	
*Regulators of apoptosis*								
B-cell leukemia/lymphoma 2 related protein A1a	Bcl2a1a		**33.896**		**5.027**		(3.713)	
Bcl2-like 1	Bcl2l1		**2.677**		(1.255)		(1.275)	
Baculoviral IAP repeat-containing 2	Birc2		(−1.428)		(1.513)		**6.288**	
Baculoviral IAP repeat-containing 3	Birc3		1.874		(1.558)		**3.599**	
Fas (TNF receptor superfamily member 6)	Fas		**11.621**		**3.261**		**6.857**	
Tnf receptor-associated factor 2	Traf2		(−1.056)		1.983		**5.107**	
*Growth factors, ligands and their modulators*								
Colony stimulating factor 1 (macrophage)		Csf1		**35.675**		**55.854**		**117.792**
Colony stimulating factor 2 (granulocyte-macrophage)		Csf2		**530**		(1.548)		(−1.436)
Colony stimulating factor 3 (granulocyte)		Csf3		**15,821.676**		**5.486**		**−31.724**
Platelet derived growth factor, B polypeptide		Pdgfb		**3.395**		(1.479)		(1.092)
*Transcription factors and regulators*								
Interferon regulatory factor 1		Irf1		**2.945**		(2.039)		**13.953**
Microphthalmia-associated transcription factor		Mitf		(−1.549)		(1.434)		**7.247**
Myelocytomatosis oncogene		Myc		**25.814**		**17.898**		(8.083)
Nuclear factor of kappa light polypeptide gene enhancer in B-cells 1, p105		Nfkb1		**4.526**		(1.599)		**3.399**
Nuclear factor of kappa light polypeptide gene enhancer in B-cells 2, p49/p100		Nfkb2		(1.368)		(1.001)		**10.255**
Nuclear factor of kappa light polypeptide gene enhancer in B-cells inhibitor, alpha		Nfkbia		**7.963**		(1.102)		(1.508)
Reticuloendotheliosis oncogene		Rel		**2.664**		(1.121)		**3.864**
Avian reticuloendotheliosis viral (v-rel) oncogene related B		Relb		(1.334)		**3.591**		**8.675**
Signal transducer and activator of transcription 1		Stat1		**2.855**		−1.257		(1.111)
Signal transducer and activator of transcription 3		Stat3		1.517		−1.104		−1.444
*Miscellaneous*								
Cyclin D1		Ccnd1		**−5.051**		**−2.667**		**−7.439**
Growth arrest and DNA-damage-inducible 45 beta		Gadd45b		**8.330**		(2.325)		**10.985**
Matrix metallopeptidase 9		Mmp9		**17.819**		(1.635)		**−5.805**

LPS stimulation increased transcription of 36 NF-κB target genes in the macrophage cells and down-regulated the expression level of one target gene. The highest up-regulated genes can be found in the groups of the cytokines/chemokines, stress response genes and growth factors. Several TFs, including TFs that take part in the NF-κB cascade, show an elevated transcription level. Previous results showed that RT-qPCR on Raw264.7 cells stimulated with *N. vitripennis* venom resulted in no significant effect in mRNA levels of NF-κB inhibitor alpha (IκBα) and A20, that are both early response genes [8]. The NF-κB Signaling Targets PCR Array resulted in one NF-κB target gene that showed down-regulated expression, and nine that were up-regulated after venom treatment. Remarkable is the up-regulation of Relb expression by venom treatment, which could possibly lead to further elevation in expression of other NF-κB target genes. On the other hand, the reporter array performed after venom treatment on HEK293T cells resulted in the induction of several TFs, like ATF2/3/4 and MEF2 (see Table 1) potentially attributing to the up-regulated expression levels shown here.

Before cells are stimulated with LPS, NF-κB subunits like p50 and p52 can already be bound to NF-κB binding sites in the promoters of a number of genes, exhibiting a certain range of expression levels [31]. After cellular stimulation with LPS, other NF-κB family members enter the nucleus and bind to those genes and to other genes, leading to enhanced gene transcription. *N. vitripennis* venom could influence this process before cells are stimulated with LPS resulting in the down-regulated expression level of NF-κB target gene, cyclin D1.

Transcription of the stress response gene, NAD(P)H dehydrogenase quinone 1 (Nqo1), is nearly 25 times up-regulated after venom treatment. This gene has been demonstrated to play an important role in protecting cells against oxidative stress [32]. Venoms of several animals, including the parasitoid wasp *Aphidius ervi*, are known to cause oxidative stress in their host organism [33,34]. The elevation of the Nqo1 gene expression after venom treatment in macrophage cells could therefore suggest a protective function. Remarkably, the venom induced transcription of two growth factors, colony stimulating factor 1 and 3 (Csf1 and Csf3) that stimulate the bone marrow progenitor cells to differentiate into macrophages or granulocytes, respectively. These proteins have an important role in innate immunity and inflammation [35]. Especially transcription of Csf1 is highly up-regulated (nearly 56 times), which even doubles when the cells are immune challenged with LPS.

However, most differentially expressed genes could be observed when venom was added to the macrophage cells, combined with an immune challenge of LPS. Transcription of two of the NF-κB target genes, tested by the NF-κB Signaling Target PCR Array, were previously tested by RT-qPCR: IκBα and interleukin 6 (IL-6) [8]. Although the suppression of IκBα transcription could not be validated in this PCR Array experiment, the 2-fold inhibition of IL-6 transcription on the other hand could be confirmed by a 1.5-fold down-regulation in the PCR Array. Transcription of the cytokine IL-1β, produced by activated macrophages and an important mediator of inflammatory response, shows a nearly 6-fold up-regulation after venom-treatment, while being 4.3 times down-regulated after co-treatment of venom and LPS. Apparently, the cells need to be immune challenged, in this case by LPS, in order for the venom to be able to suppress the inflammatory response.

Previous experiments focused on the inhibitory effect of venom on the canonical NF-κB pathway [8], in which IκB kinase beta (IKKβ) phosphorylates IκBs at *N*-terminal sites to trigger their ubiquitin-dependent degradation and induce nuclear entry of RelA:p50 dimers [36]. The remark has to be made that aside from this classical NF-κB signaling pathway, an alternative non-canonical signaling cascade has been identified that activates NF-κB signaling based on processing of the NFκB2:p100 precursor protein by IKKα [37]. In this alternative NF-κB pathway, the stimulation by LPS (among others) results in the nuclear translocation of the dimer RelB:p52 [38]. While many target genes are shared between the canonical and the non-canonical pathways, some promoters of NF-κB target genes are only recognized by RelB:p52 dimers and not by RelA:p50 dimers [31,39]. Several NF-κB target genes can bind multiple members of the NF-κB family, suggesting that they can be activated by both the canonical and the non-canonical pathways. For instance, Icam1 and Gadd45b can bind all five members of the NF-κB family (p52, p50, Rela, Relb and c-Rel) in U937 cells after LPS stimulation [31]. When Raw264.7 cells were treated with *N. vitripennis* venom, transcription of Icam1 and Gadd45b was up-regulated 17.63 and 10.99 times, respectively. Therefore, it can be suggested that *N. vitripennis* venom potentially has an inhibitory effect on the canonical pathway, but a stimulatory effect on the alternative NF-κB signaling pathway explaining the up-regulation of some inflammatory genes. This hypothesis however needs to be further investigated. Interestingly, *N. vitripennis* venom up-regulated transcription of four different NF-κB subunits when cells were co-treated with LPS, of which Nfkb2 (p52) and Relb, that take part in the non-canonical NF-κB pathway, show the highest elevations.

Interesting is the number of up-regulated NF-κB target genes involved in apoptosis regulation after venom and LPS co-treatment. Formesyn and colleagues have previously proven that serine proteases and metalloproteases in *N. vitripennis* venom cause apoptosis in non-host insect cells and suggested their possible role in immune related processes [40]. In this PCR array on murine macrophages, pro- as well as anti-apoptotic mediators were differentially expressed: Fas activation induces apoptosis [41], while Birc2, Birc3 and Traf2 are known for their anti-apoptotic effects [42,43].

An acute stimulation, for instance by LPS, is known to create two distinct waves of NF-κB recruitment to target promoters: a fast recruitment to immediately accessible promoters and a late recruitment to promoters requiring stimulus-dependent modifications in chromatin structure to make NF-κB sites accessible [44]. A relatively long (6 h) LPS-treatment was chosen in the PCR Array experiment. Unfortunately, possible transient effects caused by *N. vitripennis* venom, due to the short mRNA half-life of many of the early response genes, could not be observed by this experiment and time kinetics would need to be performed in order to have a glimpse on the complete venom profile. Next to possible time course effects, the mechanistic complexity of the venom can be considerable, since different cell lines can show large differences in their responses to specific compounds. Future experiments should therefore also incorporate different cell lines together with time kinetics in response to separate venom compounds.

Next to an NF-κB binding site, the promoter of the genes tested by this PCR array may also contain binding sites for other TFs. Instead of an up-regulated transcription by NF-κB binding to the promoter, expression of these genes can also be targeted by other TFs. When performing a TFSEARCH, the promoter sequence of a gene of interest can be screened for possible TF binding sites by correlating this sequence against the TRANSFAC MATRIX database. The upstream gene sequence 2000 nucleotides before and 50 nucleotides after the start of the gene (where the promoter sequence is assumed to be located) is inserted into the TFSEARCH program and an 85.0 threshold is used.

This was performed for five genes that showed the highest significant up-regulated fold regulation (FR) after venom-and LPS-treatment, and for four genes that had a negative FR after venom- and LPS-treatment. All nine genes obviously contained the NF-κB binding site, as the genes included in the array were previously demonstrated to be NF-κB target genes. The TF binding sites that were predicted by the program to be present in all five up-regulated genes were listed in Table 3. This means that the up-regulated transcription of these genes, next to NF-κB binding to their promoter, could also be the result of binding of these other TFs to their promoters. When looking at these TF binding sites in the promoters of the four down-regulated genes, cAMP response element-binding protein (CRE-BP) can be a possible candidate for causing the synthesis of several of the tested genes while at the same time not having an increasing effect in transcription of Csf3, Mmp9 and Ccnd1, since these genes do not contain a binding site for CRE-BP. On the other hand, a role of CCAAT-enhancer-binding proteins (C/EBPs) in the activation of NF-κB target genes can also be proposed. C/EBP binding sites can be found in promoter regions of several tested genes with an up-regulated transcription, while being absent in the promoters of some of the down-regulated genes (see Table 3). Additionally, C/EBPs can interact with p50 homodimers activating NF-κB target genes, even in the absence of canonical NF-κB activation [45]. Interestingly, the reporter array performed on HEK293T cells treated with *N. vitripennis* venom, also showed significant up-regulation of the C/EBP reporter (Table 1).

**Table 3:** TF binding sites that are commonly present in the promoters of 5 up-regulated genes tested by the NF-κB Signaling Targets PCR Array, predicted by TFSEARCH (threshold 85.0). The presence of these TF binding sites is shown for 4 down-regulated genes when cells were treated with venom and LPS. “x” represents the presence of the respective TF site in the promoter region of that particular gene. At the bottom, the fold regulations are presented for the selected genes.

NF-κB signaling target genes	Cd83	Csf1	IL15	Irf1	Icam1	Il1b	Csf3	Mmp9	Ccnd1
*TF binding site*									
NF-Kap	x	x	x	x	x	x	x	x	x
C/EBP	x	x	x	x	x	x	x	x	-
C/EBPa	x	x	x	-	x	-	x	x	-
AML-1a	x	x	x	x	x	x	x	x	x
CdxA	x	x	x	x	x	x	x	x	x
CRE-BP	x	x	x	x	x	x	-	-	-
deltaE	x	x	x	x	x	x	x	x	x
GATA-1	x	x	x	x	x	x	x	x	x
GATA-2	x	x	x	x	x	x	x	x	x
GATA-3	x	x	x	x	x	-	x	x	x
GATA-X	x	x	x	x	x	x	x	-	x
HSF2	x	x	x	x	x	x	-	x	-
MZF1	x	x	x	x	x	x	x	x	x
Nkx-2.	x	x	x	x	x	x	x	x	x
Oct-1	x	x	x	x	x	x	x	x	-
SRY	x	x	x	x	x	x	x	x	x
TATA	x	x	x	x	x	x	-	x	x
*Fold regulation*									
venom- and LPS- *vs.* LPS-treated	29.4	117.8	13.4	14.0	17.6	−4.4	−31.7	−5.8	−7.4
venom-treated *vs.* untreated	3.0	55.9	2.5	2.0	1.2	5.9	5.5	1.6	−2.7

The presence (or absence) of many other TF binding sites in specific gene promoters could possibly be an aid to explaining the apparent contradiction between the up-regulation of several inflammatory genes by *N. vitripennis* venom, but at the same time the anti-inflammatory activity of the venom on the other hand [8]. Other mechanisms can play a role in the change of gene expression: microRNAs can post-transcriptionally bind to the 3′-UTR (untranslated region) of their target mRNAs and repress protein production [46], or epigenetic phenomena like DNA methylation or histone modification can cause differential gene expression [47]. Until now, none of these processes have been investigated for the gene alterations caused by parasitoid venoms.

It needs to be commented that venom from *N. vitripennis* is a complex mixture of at least 80 different proteins. Maybe even more other venom compounds like peptides or other bio-molecules could be present, all having their own effects on gene regulation. Regarding to venomous effects on the complex apoptosis process, it would be interesting to see how the separate venom compounds, especially the serine proteases and metalloproteases as tested in insect cells [40], would affect the mammalian cell death process. However, it would also be interesting to examine what the effects of individual venom compounds would be on mammalian cells, concerning gene regulation, but also with regard to translational regulation of the NF-κB pathway.

Many of the target genes of NF-κB signaling are involved in multiple inflammatory or autoimmune diseases. Some of them are promising targets in certain therapies [48,49], while others can themselves be used as agents in processes involved in human diseases [50,51]. Interestingly, *N. vitripennis* venom significantly altered the expression of some of these possible drug targets, presenting the venom with an exciting potential as therapeutic in several diseases (Table 4). However, keep in mind that the physiological processes affected by the complex *Nasonia* venom only hint at possible interesting therapies for human disease conditions. Since until now specific mechanistic insights are lacking, further studies focusing on the affected NF-κB targets need to be performed, ideally with the responsible venom compounds.

**Table 4:** NF-κB target genes that were differentially transcribed and can be a possible drug target of *N. vitripennis* venom. Three different comparisons are presented: venom-treated *versus* untreated, venom- and LPS-treated *versus* LPS-treated cells and LPS-treated *versus* untreated. When *p* > 0.05, insignificant values are between brackets; when |FR| >2, values are in bold. (Abb = abbreviation; FR = fold regulation; Ref = reference).

Possible drug targets of venom	Abb	FR venom *versus* untreated	FR venom- and LPS- treated *versus* LPS-treated	FR LPS-treated *versus* untreated	Potential targeted diseases	Reference
NAD(P)H dehydrogenase, quinone 1	Nqo1	**24.64**	(**3.014**)	(−1.247)	acute leukemia	[52]
Cyclin D1	Ccnd1	**−2.67**	**−7.44**	**−5.05**	breast cancer	[53]
Interferon regulatory factor 1	Irf1	(**2.039**)	**13.95**	**2.95**	breast cancer	[51]
Matrix metallopeptidase 9	Mmp9	(1.635)	**−5.80**	**17.82**	cancer	[54]
Colony stimulating factor 3 (granulocyte)	Csf3	**5.49**	**−31.72**	**15,821.68**	inflammatory arthritis	[49]
Interleukin 1 beta	Il1b	**5.88**	**−4.39**	**15,647.33**	autoinflammatory diseases	[55]
Complement factor B	Cfb	(−1.007)	**−4.20**	**14.68**	complement mediated inflammatory diseases	[56]

The second highest up-regulated NF-κB target gene transcription tested after the sole addition of venom on the cells, was NAD(P)H:quinone oxidoreductase 1 (Nqo1). This enzyme detoxifies quinones and reduces oxidative stress. Low activity of this enzyme is associated with increased risk of acute leukemia in adults [52]. Inducing this detoxification enzyme in certain mammalian cell types could potentially benefit patients suffering from acute leukemia or could prevent people from developing this disease.

For the other venom targets with potential therapeutic application, the cells needed to be immune challenged with LPS, offering a possible use in diseases characterized by constitutive activity of NF-κB, like autoimmune diseases [57] or many cancers [58]. With regard to anti-inflammatory drugs, colony stimulating factor 3 (Csf3) with a nearly 32-fold decrease in transcription after venom and LPS incubation compared to LPS induction, seems to be the most interesting interaction to further investigate. This regulator of granulopoiesis has a critical role in driving joint inflammatory diseases, like rheumatoid arthritis (RA), and its antagonists may be of therapeutic value [49]. Anti-IL-1β is a commonly used drug with the name canakinumab utilized in several auto inflammatory diseases [55]. Previously mentioned inhibition of IL-1β transcription by venom- and LPS-treatment may therefore hint at a therapeutic role of *N. vitripennis* venom.

Intriguingly, the possible anti-cancer role of *N. vitripennis* venom is displayed by the regulation of different NF-κB targets. Transcription of Cyclin D1, involved in the G1-S phase transition of cells, is significantly suppressed by *N. vitripennis* venom, with and without LPS-treatment. Since pharmacological inhibition of cyclin D1/CDK4 complexes is suggested to be a useful strategy to inhibit the growth of tumors, the potential of *N. vitripennis* venom in cancer treatments may be valuable to further investigate. In contrast, the expression of Irf1 is significantly up-regulated after the co-treatment of venom and LPS, which was confirmed in the reporter array showing up-regulated transcriptional activity of the IRF1 reporter after co-treatment of venom and TNFα in HEK293T cells. A functional role of Irf1 was established in the growth suppression of breast cancer cells and it was implicated in acting as a tumor suppressor gene in breast cancer by controlling apoptosis [51]. The potential of *N. vitripennis* venom as therapeutic agent in several oncogenetic diseases is therefore interesting to look at. The possible drug targets in Table 4 show the exciting potential of *Nasonia* venom, but need to be interpreted with the necessary precaution. Future studies also require incorporate alterations at the protein level, modulations of diverse immune pathways or biological signals and determination of the exact effect of the individual responsible venom components.

## 3. Experimental Section

### 3.1. Isolation of Crude Wasp Venom

*N. vitripennis* wasps were reared on pupae of the flesh fly, *S. crassipalpis*, and maintained at 25 °C with a daily 16:8 light:dark cycle. Female wasps were allowed to host feed on flesh fly pupae for 24 h. Venom gland reservoirs were dissected into insect saline buffer (ISB) (150 mM NaCl, 10 mM KCl, 4 mM CaCl_2_, 2 mM MgCl_2_, 10 mM Hepes) [59] and centrifuged at 12,000× *g* for 10 min at 4 °C. The supernatant containing the venom was transferred to a clean microcentrifuge tube and stored frozen at −70 °C. Total protein in crude venom was determined colorimetrically at 595 nm using a Coomassie Protein Assay Reagent (No. 23200, Thermo Fisher Scientific, Rockford, IL, USA).

### 3.2. Cell Culture and Treatments

For the reporter array, human embryonic kidney cells 293T (HEK293T, kind gift from Prof. Kathleen Van Craenenbroeck, Ghent University, Ghent, Belgium) were cultured as adherent monolayers in 25 cm^2^ flasks in Opti-MEM reduced serum medium without phenol red (Thermo Fisher Scientific, Rockford, IL, USA) supplemented with 5% FBS (International Medical Products, Brussels, 1160, Belgium) and 100 U/mL penicillin (Thermo Fisher Scientific, Rockford, IL, USA) and 0.1 mg/mL streptomycin (Thermo Fisher Scientific, Rockford, IL, USA). Cells were grown in an atmosphere with 5% CO_2_ at 37 °C. Prior to the assays, cells were grown in 75 cm^2^ flasks and cultured in such a way that they are sub confluent prior to collection. The Cignal Finder™ 45-Pathway Reporter Arrays (Qiagen, SABiosciences corp., Frederick, MD, USA) consisting of 45 dual luciferase reporter assays, were used according to the manufacturer’s instructions. One hour before transfection, fresh Opti-MEM medium supplemented with 2% FBS was added to the cells and cells were placed back in the incubator. Meanwhile, DNA reporter constructs were first dissolved in 50 μL/mL Opti-MEM and incubated for 5 minutes at room temperature. Subsequently, PEI transfection reagent (Thermo Fisher Scientific, Rockford, IL, USA) was diluted in Opti-MEM without serum or antibiotics, and 50 μL/well was dispensed into 96-well white tissue-culture plates. For reverse transfection, freshly grown cells were counted and adjusted to 1.4 million cells/mL in Opti-MEM containing 2% FBS and 1% non-essential amino acids (NEAA, Thermo Fisher Scientific, Rockford, IL, USA), without antibiotics. Cells (50 μL; 7 × 104 cells/well) were added to each well and incubated overnight at 37 °C with 5% CO_2_. Transfection media were removed 6–8 h after transfection and replaced with 100 μL fresh Opti-MEM, supplemented with 5% FBS and 1% NEAA, and incubated again overnight at 37 °C. One hour prior to venom induction, media were removed and replaced with fresh Opti-MEM supplemented with 0.5% FBS and 1% NEAA. Cells (two array plates) were then induced with *N. vitripennis* venom at a concentration of 2.5 μg/mL for 8 h, the other 2 plates were treated with ISB and served as controls. Each plate contains a duplicate of the reporter.

For the PCR array, mouse macrophage-like Raw264.7 cells (kind gift from Prof. Kathleen Van Craenenbroeck, Ghent University, Ghent, Belgium) were maintained in RPMI 1640 medium (Thermo Fisher Scientific, Rockford, IL, USA) at 37 °C in 5% CO_2_ humidified air. Medium was supplemented with 10% FBS, 100 U/mL penicillin and 0.1 mg/mL streptomycin. Twenty-four hours before induction, cells were seeded in multiwell dishes so that they were confluent at the time of the experiment. Raw264.7 cells were submitted to the following different treatments: untreated; 6-h LPS induction (1 µg/mL); 6 h and 15 min incubation with 10 µg/mL of *N. vitripennis* venom; 6 h LPS induction and 6 h and 15 min venom incubation. The 4 treatments were performed in duplicate.

### 3.3. Reporter Array Analysis

Dual-luciferase reporter activity was determined using a dual-luciferase reporter assay system (Promega, Madison, WI, USA), following the manufacturer’s instructions using a Victor 3TM 1420 Multilabel Counter plate reader (PerkinElmer, Waltham, MA, USA). Induced TFs were reported as luminescence ratios by dividing the Firefly signal by the Renilla signal. Subsequently, normalization was performed using the ratio of control wells on every plate (negative and positive controls). Data was evaluated by Student’s *t*-tests and Mann-Whitney *U* tests. A value of *p* < 0.05 was considered statistically significant. All statistical analysis were performed with Prism 5.0 (GraphPad Software, Inc., La Jolla, CA, USA, 2011).

### 3.4. Total RNA Extraction and Reverse Transcription

RNA was isolated by using TRIzol Reagent (Thermo Fisher Scientific, Rockford, IL, USA) as described previously [60]. An on-column DNase digestion was performed and the concentration of RNA in all 8 samples was measured. Equal amounts of RNA were reverse-transcribed into cDNA using the RT^2^ First Strand Kit (Qiagen, Frederick, MD, USA) following the manufacturer’s instructions.

### 3.5. Real-Time PCR-Based Array Analysis

The relative expression of NF-κB signaling target genes was determined in each of the 8 samples by RT-qPCR (RT^2^ Profiler Mouse NF-κB Signaling Targets PCR Array; SABiosciences Corp., Frederick, MD, USA) using the qPCR master mix (RT^2^ SYBR Green; SABiosciences Corp., Frederick, MD, USA) according to the supplier’s directions. Mixes were pipetted into 384-well PCR array plates to evaluate the expression of 84 NF-κB signaling target genes. RT-qPCR was performed in technical duplicates (Roche LightCycler 480, 384-well block, Mannheim, Germany). Raw data from the real-time PCR were uploaded using a PCR array data analysis template available at RT^2^ Profiler PCR Array Data Analysis version 3.5 (http://www.sabiosciences.com/pcr/arrayanalysis.php). Quality controls included within the array plates confirmed the lack of DNA contamination and successfully tested for RNA quality and PCR performance. The integrated Web-based software package for the PCR array system automatically performed all comparative threshold cycle (ΔΔCt)-based fold-change calculations from the uploaded data. For these calculations, the average expression of 3 housekeeping genes (β-actin, glyceraldehydes-3-phosphate dehydrogenase and β2-microglobulin) was used for normalization of the data. After normalization, the relative expression of each gene was averaged for the 2 samples in each condition. Fold regulations in average gene expression were expressed as the difference in expression of venom-treated compared with untreated cells, or of venom- and LPS-treated compared with only LPS-treated cells. A fold change ≥2.5 with *p* ≤ 0.05 was considered significant.

## 4. Conclusions

By using high-throughput screening tools such as the reporter and PCR arrays performed here, the multi-facetted effects of venom can become visible, pinpointing interesting pathways and targets for further investigation. The effect of *N. vitripennis* venom on 45 intracellular signaling cascades was analyzed with a commercially available reporter array in HEK293T cells either immune challenged with TNFα or not. Interesting pathways were affected, of which several are related with an early stress response and others that need to be activated by steroid compounds. Whether steroids, possibly present in the venom, induced these reporters or venom stimulation contributes to the release of steroids in cells, still needs to be further investigated. Since most of the affected pathways are involved in multiple biological processes, more detailed research needs to be performed on both transcript and protein level in order to unravel how the venom affects these processes. In addition, the possible protein interactions with other TFs might be investigated, because the composition of the TF complexes influences their promoter specificity and hence their target pathways. Furthermore, other induction time points or venom concentrations may also provide useful information. The NF-κB Signaling Target PCR Array performed on Raw264.7 macrophages treated with *N. vitripennis* venom and/or LPS uncovered several new ideas on how the venom exerts its complex effects. Interestingly, the proinflammatory cytokine IL-1β was significantly suppressed after the venom and LPS co-treatment, indicating the anti-inflammatory action of the *Nasonia* venom. Previous data describing the inhibition of the canonical NF-κB pathway by the venom [8], is not that obvious when looking at the expression of a large number of NF-κB target genes. Several aspects encourage us to be cautious in interpreting the results. The complexity of the venom mixture applied on the cells creates multiple crosstalk effects that could be circumvented when working with separate venom compounds. The fact that one time-point was used, only gives a glimpse of the complete picture. Additionally, changes in transcription expression do not always translate into the same changes at protein level, since alterations in translation efficiency and post-translational modifications can lead to a different end-result. Possible effects on the non-canonical NF-κB pathway next to the canonical pathway, in addition to the presence of multiple TF sites in the promoter of NF-κB target genes, make the puzzle more difficult to solve. However, keeping all these remarks in mind, still several interesting hints for future research could be noted, which was the original intent of this experiment. Some NF-κB target genes that were differentially expressed by the addition of *N. vitripennis* venom, with or without LPS-treatment, are drug targets of major diseases, hinting to possible future biomedical application as therapeutic agent in several human diseases. Performing time kinetics and dose-responses by the separated venom compounds seems the logical next step.

## Supplementary Materials

Supplementary materials can be accessed at: http://www.mdpi.com/2072-6651/7/6/2051/s1.
