# Supplementary material for: Exploring the Potential of Venom from Nasonia vitripennis as Therapeutic Agent with High-Throughput Screening Tools
Source: Toxins (Basel). 2015 Jun 3;7(6):2051–70. doi: 10.3390/toxins7062051 (PMC4488689; doi:10.3390/toxins7062051)
Supplement: Supplementary file 1 [file toxins-07-02051-s001.pdf]

Supplementary Information

Table S1. Effect of *N. vitripennis* venom in Raw264.7 cells, either induced with lipopolysaccharide (LPS) or not, on NF-κB signaling targets. Fold regulation of all tested NF-κB signaling target genes are presented for 3 different comparisons. When $p > 0.05$, insignificant values are between brackets; when |FR| > 2, values are in bold. (Abb = abbreviation; FR = fold regulation).

NF-κB signaling target genes	Abb	FR LPS-treated *versus* untreated	FR venom-treated *versus* untreated	FR LPS- and venom-treated *versus* LPS-treated
Cytokines/chemokines and their modulators				
Chemokine (C–C motif) ligand 12	Ccl12	(20,270)	(1,162)	(−7,732)
Chemokine (C–C motif) ligand 22	Ccl22	**917,635**	(1,032)	(−1,532)
Chemokine (C–C motif) ligand 5	Ccl5	**1254,881**	(2,107)	(1,750)
Chemokine (C–C motif) receptor 5	Ccr5	**1,625**	(1,875)	(2,346)
Chemokine (C–X–C motif) ligand 1	Cxcl1	(−1,677)	(1,387)	(43,365)
Chemokine (C–X–C motif) ligand 10	Cxcl10	**483,835**	(2,254)	(−1,025)
Chemokine (C–X–C motif) ligand 3	Cxcl3	**111,806**	(3,484)	(34,595)
Chemokine (C–X–C motif) ligand 9	Cxcl9	(−8,667)	(−6,047)	(1,567)
Interferon beta 1, fibroblast	Ifnb1	(78,387)	(2,645)	(−1,304)
Interferon gamma	Ifng	(−1,148)	(1,387)	(8,926)
Interleukin 12B	Il12b	(7,387)	(1,387)	(7,620)
Interleukin 15	Il15	(3,325)	(2,527)	**13,408**
Interleukin 1 alpha	Il1a	**1273,082**	(2,414)	(−1,242)
Interleukin 1 beta	Il1b	**15647,327**	**5,885**	**−4,392**
Interleukin 1 receptor, type II	Il1r2	(−1,148)	(1,387)	(1,741)
Interleukin 1 receptor antagonist	Il1rn	**26,052**	(3,074)	(−1,145)
Interleukin 2	Il2	(−1,148)	(1,387)	(1,741)
Interleukin 2 receptor, alpha chain	Il2ra	(−1,148)	(1,387)	(1,778)
Interleukin 4	Il4	(−1,556)	(1,405)	(1,741)
Interleukin 6	Il6	**525,452**	(2,419)	(−1,272)

Table S1. *Cont.*

**NF-κB signaling target genes**	**Abb**	**FR LPS-treated *versus* untreated**	**FR venom-treated *versus* untreated**	**FR LPS- and venom-treated *versus* LPS-treated**
Lymphotoxin A	Lta	**25,056**	(3,037)	(1,094)
Lymphotoxin B	Ltb	(1,733)	(1,012)	(1,044)
Tumor necrosis factor	Tnf	**36,399**	(−1,244)	−1,193
Tumor necrosis factor (ligand) superfamily, member 10	Tnfsf10	(7,341)	(1,049)	(1,708)
Immunoreceptors				
CD40 antigen	Cd40	**68,505**	**9,933**	(4,098)
CD80 antigen	Cd80	**3,113**	(2,621)	(2,723)
CD83 antigen	Cd83	(3,545)	(2,949)	**29,395**
Myeloid differentiation primary response gene 88	Myd88	(1,082)	(−1,117)	(2,514)
Tumor necrosis factor receptor superfamily, member 1b	Tnfrsf1b	**22,445**	(1,469)	(1,250)
Proteins involved in antigen presentation				
Complement component 3	C3	**3,651**	(2,035)	(1,175)
Complement factor B	Cfb	**14,677**	(−1,007)	**−4,199**
Transformation related protein 53	Trp53	(−1,881)	(1,000)	(1,521)
Cell adhesion molecules				
Intercellular adhesion molecule 1	Icam1	(1,405)	(1,212)	**17,631**
Selectin, endothelial cell	Sele	(2,441)	(1,265)	(978,229)
Selectin, platelet	Selp	(−8,889)	(−4,706)	(1,741)
Vascular cell adhesion molecule 1	Vcam1	(−1,148)	(1,387)	**3,481**

Table S1. *Cont.*

**NF-κB signaling target genes**	**Abb**	**FR LPS-treated *versus* untreated**	**FR venom-treated *versus* untreated**	**FR LPS- and venom-treated *versus* LPS-treated**
Acute phase proteins				
Angiotensinogen (serpin peptidase inhibitor, clade A, member 8)	Agt	(7,806)	(1,387)	(−5,149)
Complement component 4A (Rodgers blood group)	C4a	(−1,595)	(1,051)	(1,270)
Coagulation factor III	F3	**41,407**	(4,466)	(−1,979)
Plasminogen activator, urokinase	Plau	(−4,608)	(−2,407)	(12,884)
Stress response genes				
NAD(P)H dehydrogenase, quinone 1	Nqo1	(−1,247)	**24,637**	(3,014)
Prostaglandin-endoperoxide synthase 2	Ptgs2	**698,790**	(1,715)	(−1,605)
Superoxide dismutase 2, mitochondrial	Sod2	**4,919**	−1,052	(1,301)
Cell surface receptors				
Epidermal growth factor receptor	Egfr	(42,516)	(1,387)	(−28,043)
Regulators of apoptosis				
B-cell leukemia/lymphoma 2 related protein A1a	Bcl2a1a	**33,896**	**5,027**	(3,713)
Bcl2-like 1	Bcl2l1	**2,677**	(1,255)	(1,275)
Baculoviral IAP repeat-containing 2	Birc2	(−1,428)	(1,513)	**6,288**
Baculoviral IAP repeat-containing 3	Birc3	1,874	(1,558)	**3,599**
Fas (TNF receptor superfamily member 6)	Fas	**11,621**	**3,261**	**6,857**
Fas ligand (TNF superfamily, member 6)	Fasl	(−1,148)	(1,387)	(1,741)
Tnf receptor-associated factor 2	Traf2	(−1,056)	1,983	**5,107**
X-linked inhibitor of apoptosis	Xiap	(1,136)	(1,368)	(1,508)

Table S1. *Cont.*

NF-κB signaling target genes	Abb	FR LPS-treated *versus* untreated	FR venom-treated *versus* untreated	FR LPS- and venom-treated *versus* LPS-treated
Growth factors, ligands and their modulators				
Colony stimulating factor 1 (macrophage)	Csf1	**35,675**	**55,854**	**117,792**
Colony stimulating factor 2 (granulocyte-macrophage)	Csf2	**530,000**	(1,548)	(−1,436)
Colony stimulating factor 3 (granulocyte)	Csf3	**15821,676**	**5,486**	**−31,724**
Platelet derived growth factor, B polypeptide	Pdgfb	**3,395**	(1,479)	(1,092)
Early response genes				
Early growth response 2	Egr2	(2,222)	(1,050)	(4,104)
Transcription factors and regulators				
Interferon regulatory factor 1	Irf1	**2,945**	(2,039)	**13,953**
Microphthalmia-associated transcription factor	Mitf	(−1,549)	(1,434)	**7,247**
Myelocytomatosis oncogene	Myc	**25,814**	**17,898**	(8,083)
Nuclear factor of kappa light polypeptide gene enhancer in B-cells 1, p105	Nfkb1	**4,526**	(1,599)	**3,399**
Nuclear factor of kappa light polypeptide gene enhancer in B-cells 2, p49/p100	Nfkb2	(1,368)	(1,001)	**10,255**
Nuclear factor of kappa light polypeptide gene enhancer in B-cells inhibitor, alpha	Nfkbia	**7,963**	(1,102)	(1,508)
Nuclear receptor subfamily 4, group A, member 2	Nr4a2	(−1,847)	(−1,307)	(2,193)
Reticuloendotheliosis oncogene	Rel	**2,664**	(1,121)	**3,864**
V-rel reticuloendotheliosis viral oncogene homolog A (avian)	Rela	(−1,172)	(1,619)	(3,266)
Avian reticuloendotheliosis viral (v-rel) oncogene related B	Relb	(1,334)	**3,591**	**8,675**
Signal transducer and activator of transcription 1	Stat1	**2,855**	−1,257	(1,111)
Signal transducer and activator of transcription 3	Stat3	1,517	−1,104	−1,444
Signal transducer and activator of transcription 5B	Stat5b	(−1,904)	(1,171)	(3,300)

Table S1. *Cont.*

**NF-κB signaling target genes**	**Abb**	**FR LPS-treated *versus* untreated**	**FR venom-treated *versus* untreated**	**FR LPS- and venom-treated *versus* LPS-treated**
Enzymes				
Matrix metallopeptidase 9	Mmp9	**17,819**	(1,635)	**−5,805**
Aldehyde dehydrogenase family 3, subfamily A2	Aldh3a2	(−1,811)	(1,905)	(1,561)
Miscellaneous				
Cyclin D1	Ccnd1	**−5,051**	**−2,667**	**−7,439**
Cyclin-dependent kinase inhibitor 1A (P21)	Cdkn1a	(1,238)	(1,173)	(1,949)
Coagulation factor VIII	F8	(1,278)	(2,139)	(3,196)
Growth arrest and DNA-damage-inducible 45 beta	Gadd45b	**8,330**	(2,325)	**10,985**
